# Application of the So-Called New Wartime Antiseptics to the Several Branches of the Surgery of Civil Life*Read before the Seventh District branch of the Medical Society of the State of New York, Rochester, N. Y., September 28th, 1916.

**Published:** 1916-11

**Authors:** Charles W. Hennington

**Affiliations:** Rochester, N. Y.


### BUFFALO MEDICAL JOURNAL

Yearly Volume 72 NOVEMBER, 1916 Number 4

### ORIGINAL ARTICLES

The right is reserved to decline papers not dealing with practical medical and surgical subjects, and such as might offend or fail to interest readers. Contributors are solely responsible for opinions, methods of expression and revision of proof.

---

### Application of the So-called New Wartime Antiseptics to the Several Branches of the Surgery of Civil Life.*

CHARLES W. HENNINGTON, Rochester, N. Y.

Since the great war began, hypochlorites have come to their own as surgical antiseptics. As yet they are still intimately associated with military surgery. But the tremendous opportunity for their study has so absolutely established the method of treatment that it must be adopted in all wounds of civil life in any way analogous. Besides this, the time has come to test out the hypochlorites in other conditions not entirely analogous. But further and in addition to these obvious extentions, I have believed that it would be worth while to test them out in the various branches of surgery. They may prove of value in many other conditions in which antiseptics are used. Perhaps many unusual uses will be found for them to the advantage of all departments of surgical effort.

The most widely discussed of these hypochlorites is sodium hypochlorite, often known as Dakin's fluid, which has been introduced since the war began. Its use in wounds has been thoroughly investigated by Carrel at the Rockefeller laboratory and hospital at Campiegne. It is now used very extensively along the entire western front. Of late it has been used by the Germans as well, having been highly recommended by the great German surgeon von Bruns.

Besides sodium hypochlorite I wish to include those of potassium, calcium, and the recent very complex synthetic hypochlorites. These later hypochlorites such as para-toluene-

---

*Read before the Seventh District branch of the Medical Society of the State of New York, Rochester, N. Y., September 28th, 1916.

sodium-sulphochloramide, known in England as chloramine and in the United States as chlorazene, were also developed by Dakin and others in the search for more stable and less irritating forms. It does not seem unlikely that one of these complex hypochlorites may ultimately come into general use.

Besides these various hypochlorites, the war has called attention to valuable properties possessed by Sir Almoth E. Wright's solution of 5% saline and 0.5% sodium citrate used generally as a wet dressing. He had introduced this solution several years before the war, but again made extensive studies in its properties at his station at Boulogne. Wright's solution has much to commend it. It has gained a definite place in the treatment of infected wounds. My personal experience with Wright's solution has extended over about five years. Even if Wright's explanation of lymph lavage is not acceptable, yet it does seem that such a hypertonic salt solution would produce an osmotic flow toward the dressings and that the sodium citrate would prevent the coagulation of discharges. However it lacks the direct antiseptic properties of the hypochlorites which seem to be displacing it.

The hypochlorites and their disinfectant properties have been known over a century. Dakin himself has taken very particular pains to give the history. His so-called "new antiseptic" was discovered in 1788 by Berthollet. The composition of the mixture was not known precisely at the time. It was used as a disinfectant and a bleacher. It was produced by the action of chlorin on aqueous alkali solutions, especially soda. In 1792 the corresponding potassium salts were made commercially at the Javel works near Paris. This Javel water was used as a disinfectant. In 1820 the solution first mentioned was prepared in another way and became known as Labarraque's disinfectant fluid. Still later these two fluids were replaced largely by the solid and somewhat more stable so-called chloride of lime bleaching powder, more correctly known as chlorinated lime. It is a combination of calcium chloride and hypochlorite.

Dakin prepares his sodium hypochlorite solution from chlorinated lime and washing soda. His fluid is essentially similar to the first substance introduced over a century and a quarter ago. The disinfecting properties of all these substances were known but surgeons were hampered by their sharply irritating alkaline properties when in contact with tissues. It should be recalled that Semmelweis in Vienna in 1846 used chlorinated lime to eradicate puerperal fever from his clinic. Surgeons have widely used chlorinated lime and soda to sterilize the hands before operations. In the last decade "Chloride of lime" has been used widely to purify municipal water supplies, and water in tanks and wells. The English expedition in Arabia is purifying Tigris River water in this way following the method of Major Lyster of the U. S. A. M. C.

The action of "chloride of lime" depends upon the liberation of chlorine. The commercial form will give about 35% available chlorine. In water it is effective in the proportion of two parts of chlorine to 1,000,000 of water. The powder is not added directly but is first dissolved and the supernatent liquid only is used.

A quite recent use is in the artificial purification of oysters. Only one part available clorine to 4,000,000 parts of water and repeated in six hours is required.

It is not remarkable, therefore, that such a group of substances, of this history and of so wide present-day acceptance, should find a field of usefulness in the treatment of wounds. The originality of Dakin's fluid consists in depriving the sodium hypochlorite of its irritating properties due to the presence of free caustic alkali.

The principle of the preparation advocated by Dakin is as follows:—Chloride of lime (bleaching powder, chlorinated lime) is decomposed with a solution of sodium carbonate and to the filtered solution containing sodium hypochlorite is added boric acid to neutralize the excess of alkali. The resultant solution is a balanced mixture of hypochlorite and polyborates of sodium with small amounts of free hypochlorous acid and boric acid.

There seem to be several methods of preparing Dakin's fluid. They are as follows:

1. Dissolve 140 grams of dry sodium carbonate or 400 grams of the washing soda crystals in 10 litres of tap water. Add 200 grams of "chloride of lime" (chlorinated lime) of good quality and shake well.

In half an hour the clear liquid is siphoned off from the precipitate of calcium carbonate and filtered through a plug of cotton.

Then add 40 grams of boric acid merely to keep it neutral, and the solution is ready for use. It contains 0.5 to 0.6% of sodium hypochlorite. It does not keep longer than one week.

1a. Dissolve 105 grams of dry sodium carbonate in 1 litre of water.

Add 150 grams of "chloride of lime." The mixture is filtered and a measured portion is rapidly titrated with a boric acid solution of known strength, using phenolphthalein suspended in water as indicator, in order to determine the amount of solid boric acid to be added to the rest of the filtrate. It is best to add slightly less boric acid than the estimated amount because any excess is to be avoided. The solution thus prepared is much stronger, containing about

4% of sodium hypochlorite. It must be diluted with six parts of water before using. It will keep for one month however.

2. Carrel's Modification.

Dissolve 200 grams chlorinated lime in 5 litres of water, agitate thoroughly, and set aside over night, in a 12 litre jar. Dissolve 100 grams dry sodium carbonate and 80 grams soda bicarbonate in 5 litres of water.

Pour the two together and vigorously agitate one minute. In half an hour the clear liquid is siphoned off and filtered through paper. Then test with phthalein powder that it is not alkaline.

This newer method is more generally used now. It is about .5% strength and therefore ready for use.

Now in order to consider in what conditions, sodium hypochlorite or similar hypochlorites may be found useful, it is important that we first discuss the principles of their use in wounds. By doing so thoroughly it will be apparent how these principles might be transferred to conditions other than ordinary wounds. It is elementary, of course, that the hypochlorites should not be used with other substances as it decomposes many, notably alcohol, ether, peroxide, and various antiseptics. It will be recalled that in the tests for alkalinity the phenolphthalein is used as a powder or an aqueous solution for the alcoholic solution would be attacked.

Fortunately Dakin's solution is very cheap for experience has shown that large quantities should be used in wounds and the remotest parts irrigated. The wounds should be laid open wide with free incision to provide good drainage. Small tubes should be placed in the recesses and the irrigations repeated every two hours by injecting about 10 c.c. into each tube. The solution will thus bathe the deeper parts of the wound and is allowed to escape into the soft wet dressings, which should be changed frequently. Or it may drain into a trough conveniently placed under the wounded extremity. As much as 1 or 2 litres daily is used, or continuous drop by drop irrigation may be instituted for several days or a week. In this case the skin is to be protected with vaseline. If the wound is entirely superficial it can be immersed in a bath of the solution, but as a rule irrigation is more effective. There should be conscientious intelligent attention given to all details. Packing the wound with wet gauze or with sacs of agar to distend it has been found very useful. The deep wound can thus be converted so to speak into a large flattened-out surface wound.

The hypochlorites due to their action on the N. H. group dissolve necrotic tissue and discharges. Osmosis occurs. Deodorization is achieved. Granulation is promoted.

An ideal antiseptic is one which can destroy bacteria without destroying the elements of the tissues. Protection of the cells "orthophylaxy" (Delbet) must be in mind constantly. Sodium hypochlorite possesses these advantages as well as permeability, ability to penetrate, solubility. It is non-toxic, thus much more desirable than the metallic antiseptics as mercury, silver, etc. It forms no insoluable compounds with proteid as is done by these and phenol and iodine. It has higher germicidal value than iodine and the phenol derivatives.

There are but two disadvantages. First, it may irritate the tissues if alkali is present, due to improper preparation or to decomposition. Second, it does not keep long, decomposing in a week in the diluted form and in a month in the concentrated form. The newer complex synthetic hypochlorites such as chloramine referred to above, are entirely stable and not so irritating. They also have another very important advantage being more easy to use for they are stable powders which merely require solution. Or they can be used dry. In passing it may be mentioned that some military surgeons do use dry dressings in conjunction with irrigations. Lumiere and others speak of using a mixture of calcium chloride and boric acid 1 to 3. Others use a much more diluted mixture 1 to 10. Dakin speaks of packing the wound with gauze impregnated with the chloramine. Chloramine is so far from being toxic or irritating that it is very useful in wounds of the jaw and in the mouth as a mouth wash.

Truly remarkable results have been achieved in military surgery by the use of these substances. In wounds of the soft tissues and in compound fractures and in many compound joint wounds the method is so absolutely established that it ought to be transferred to similar conditions arising in civil life. It seems but a step to apply it to the infected extremities that we meet so frequently in our dispensary practice; likewise in the treatment of persistent sinuses and fistulas; so also in leg ulcers and in dressing gangrenous extremities; in all open suppuration and ulceration; in burns; in plastic surgery to prevent infection. These and other conditions seem rather closely related to the types in which the treatment has been found so successful.

The hypochlorites might be tried out in persistent empyema and other sinuses and in urinary and gall bladder fistulas. Chloramine has been used in the mouth and urinary bladder in 0.5% strength for irrigation, and for chronic urethritis in 2% strength. In wounds it is usually used in 4% concentration. Might it not be useful in pyorrhea; in infections associated with vaginal discharge; in chronic inflammatory discharging conditions of the middle ear, nose, eye? I dare mention but a few of these as types.

In my experience I have used the hypochlorites in instances closely related to the group of various open lesions in which their use is based entirely upon analogy. But the further group of possibilities offers a large field as yet unexplored for I have found nothing in the literature. We know however that the developments of one field may often be appropriated advantageously to other remote fields of endeavor.

633 Park Avenue.

---

**Salts and Iron in Modified Cow's Milk.** Bartlett, Arch. of Paediatrics, Jan., calls attention to the fact that while salts are present to a greater degree in cow's milk than in human milk, the dilution of the former, reduces them too much. A liter of human milk contains 1-4 m.g. of oxid of iron, of cow's milk 0.4-0.7. Hence cow's milk as ordinarily diluted, is liable to produce anaemia, although the infant possesses stores of iron in the liver and spleen sufficient on breast feeding to carry it over till iron is obtained from extrinsic foods. The deficit of iron usually shows itself at the sixth or seventh month on artificial feeding, at the same time that dentition produces more or less disturbance. Egg yolk, cereals, cooked fruit, cooked green vegetables, especially spinach are recommended as additions to the diet, serving also to prevent scurvy (Barlow's disease). Carter, Practitioner, Feb., with special reference to UNDILUTED CITRATED MILK, incidentally covers a point of prophylaxis of infantile anaemia. Milk thus treated does not coagulate in large masses and hence may be given undiluted. The relative diminution of bulk of food imposes less strain upon the digestive organs, and especially tends to prevent gastric dilatation. 1/10 of the weight of the infant per day is the approximate standard suggested by Budin but the amount should be checked according to the condition of the individual child. Feedings should be as far apart as possible. (Note: We have been impressed in the last few years with the very general interest taken by young mothers in infant hygiene, their scrupulous care in following dietetic directions and the very practical results in the way of ''good babies'' and relative freedom of the mothers, due to careful feeding at intervals approximately double those in vogue a generation ago).

---

**Mercuric Succinimid in Poliomyelitis.** Large doses, intramuscularly, are recommended by Barton L. Wright, U. S. N., quoted in Med. Herald, Sept.

